# Preliminary Data on the Safety of Phytoene- and Phytofluene-Rich Products for Human Use including Topical Application

**DOI:** 10.1155/2018/5475784

**Published:** 2018-04-15

**Authors:** Fabien Havas, Shlomo Krispin, Antonio J. Meléndez-Martínez, Liki von Oppen-Bezalel

**Affiliations:** ^1^IBR (Israeli Biotechnology Research) Ltd., 8122503 Yavne, Israel; ^2^Department of Nutrition & Food Science, Universidad de Sevilla, 41012 Seville, Spain

## Abstract

The colorless carotenoids phytoene and phytofluene are comparatively understudied compounds found in common foods (e.g., tomatoes) and in human plasma, internal tissues, and skin. Being naturally present in common foods, their intake at dietary levels is not expected to present a safety concern. However, since the interest in these compounds in the context of many applications is expanding, it is important to conduct studies aimed at assessing their safety. We present here results of in vitro cytotoxicity and genotoxicity studies, revealing no significant cytotoxic or genotoxic potential and of short- and long-term human in vivo skin compatibility studies with phytoene- and phytofluene-rich tomato and* Dunaliella salina* alga extracts, showing a lack of irritancy or sensitization reactions. These results support the safe use of phytoene- and phytofluene-rich products in human topical applications.

## 1. Introduction

Dietary carotenoids are important compounds in the context of food science, nutrition, and health as they account for the color of many foods, some can be converted into compounds exhibiting vitamin A activity, and there is a large body of evidence indicating that they may exert health-promoting actions and contribute to reducing the risk of developing several diseases [1, 2]. Their value as food colorants, components of functional foods, and related products such as dietary supplements and other over-the-counter healthcare products is therefore undeniable. There is also an expanding interest in these compounds in the context of nutricosmetics, a growing sector that includes products at the intersection between food, nutrition, health, and cosmetics. These can promote “beauty from within” and are usually referred to as “beauty foods,” “beauty pills,” or “oral cosmetics” [3, 4]. Similarly, carotenoids have a long history of use in topical cosmetic formulations, aimed at delivering diverse beauty and personal care benefits like antiaging, anti-inflammation, antioxidation, and more [5–7].

Historically, the most studied carotenoids (and those most often used in carotenoid-linked health- and food-related claims) have been colored carotenoids such as lycopene, *β*-carotene, astaxanthin, or lutein. A relatively wide array of safety data is available regarding each of these compounds, as will be discussed later.

This is not the case for phytoene and phytofluene, which are considered the precursors of all colored carotenoids [8] and are rarities among carotenoids as they are colorless. This can explain in part the fact that phytoene and phytofluene have not been studied as intensively as other dietary carotenoids despite being present in widely consumed foods (tomatoes, citrus, carrots, among others) [9]. Besides, phytoene and phytofluene have been shown to be efficiently released from diverse food matrices using simulated* in vitro* digestions [10]; phytoene and phytofluene are present in human plasma, internal tissues, and skin [11–14]; and, more importantly, there are studies pointing to the fact that phytoene and phytofluene may be involved in diverse health-promoting actions. More specifically, phytoene and phytofluene could participate in antioxidant [15, 16] and anti-inflammatory actions [17, 18], decrease DNA damage in lymphocytes [19], and protect against skin and other types of cancer (leukemia, breast, endometrium, and prostate) [20–24] and against light-induced erythema in the skin [25]. Interestingly, these colorless carotenoids could also participate in biological actions leading to cosmetic benefits. Thus, it has been recently reported that a 12-week intervention with a freeze-dried natural tomato powder food supplement (Israeli Biotechnology Research Ltd. (IBR), Yavne, Israel) led to improvements in a series of skin quality parameters (skin radiance, suppleness, evenness, smoothness, moisturization, elasticity, visible skin health, visible skin youthfulness, and overall skin beauty) that were both clinically assessed and self-perceived by the volunteers [25]. The growing importance of the scarcely studied carotenoids phytoene and phytofluene across several research fields has been the subject of a dedicated comprehensive review [26].

Being naturally present in a variety of common and exotic dietary sources [26] their intake at dietary levels is not expected to pose important safety concerns. However, since their interest in the context of the diet, functional foods, and nutricosmetics is expected to expand considerably in the near future; and, considering the relative scarcity of data on this matter, it is important to conduct studies aimed at assessing their safety.

Following the above, we thus present herein results of several* in vitro* and topical* in vivo* human toxicological studies with additional phytoene- and phytofluene-rich liquid extracts from tomato and algal sources.

## 2. Materials and Methods

### 2.1. *In Vitro* Toxicology: Cytotoxicity by Neutral Red Release (NRR) Protocol

#### 2.1.1. Phytoene- and Phytofluene-Rich Products Tested

Tested substances (Israeli Biotechnology Research Ltd. (IBR), Yavne, Israel) consisted of phytoene- and phytofluene-rich botanical extracts in oil carriers: (1) a tomato extract in squalene oil, containing ca. 0.75 mg/mL phytoene and phytofluene, and (2) an extract of* Dunaliella salina* alga in hydrogenated polydecene oil, containing ca. 1 mg/mL phytoene and phytofluene (both by UV spectrophotometry). Paraffin oil (aka mineral oil, Fluka) was used as negative control, as well as to dilute tested substances. An SDS solution (0.01, 0.05, and 0.20%) served as positive control.

#### 2.1.2. Experimental Conditions

The methodology was adapted from that described elsewhere [27]. The tested substances above were diluted and applied to a monolayer of rabbit cornea fibroblasts (SIRC #2-552, CCL60; ATCC, Virginia USA) marked with a vital dye (Neutral Red). The cells were cultured in complete DMEM medium supplemented with decomplemented fetal calf serum and a penicillin/streptomycin solution, at 37°C under 5% CO_2_. Cells were seeded and incubated for 24 h, the culture medium was removed, and Neutral Red coloring solution was added. After 3 h further incubation, the coloring solution was removed and replaced by complete culture medium. The cells were then maintained at room temperature for 30 min, following which the wells were washed with PBS and treated with the diluted test products. Following 60 seconds' contact, the wells were washed with PBS. Acetic acid (in ethanol) was added, and after 15 min agitation the revealed solutions were transferred to 96-well plates (in duplicate) and optical density was read (Multiskan RC ascent 2.6 reader). The percentage of cell death was calculated as follows: % cell death = 100 × (OD of treated wells/OD of control wells). The tested substances were tested in dilutions at 5, 15, 25, 35, and 50%. The IC50 of each test item was then calculated by linear regression.

The results are then classified as described in Table 1.

### 2.2. *In Vitro* Toxicology: Genotoxic Potential

#### 2.2.1. Phytoene- and Phytofluene-Rich Products Tested

The tested substance consisted of a hydrophobic phytoene- and phytofluene-rich* Dunaliella salina* extract (Israeli Biotechnology Research Ltd. (IBR), Yavne, Israel) in hexane, which was evaporated and then taken up in a like volume of ethanol and diluted 1/10 to 1/10000 with ultrapure water.

#### 2.2.2. Experimental Conditions

The test method is part of a cell-free* in vitro* reconstitution of the repair process of DNA damage generated by Reactive Oxygen Species (ROS) [28], where highly purified plasmid DNA adsorbed onto sensitized wells were exposed for 30 min to the potential genotoxic agent and then repaired by exposure over 3 h to repair enzymes and dUTP-biotin, resulting in the incorporation of biotin moieties. These were then tagged by 15-minute exposure to streptavidin and a chemiluminescent peroxidase substrate (5 min), and the luminescence was quantified with a luminometer (SFRI Lumax 2).

The genotoxicity of the sample was evaluated according to the quantity of repairs thus detected (directly related to the amount of damage caused), as expressed by the ratio of luminescence obtained with the test sample to that of the solvent control. A repair ratio greater than 2 would indicate significant genotoxicity, while a ratio of less than 2 would indicate no significant genotoxicity. Methyl methanesulfonate (MMS) was used as a positive control.

### 2.3. Topical Toxicology* In Vivo* (Human): 48 h Patch Testing

#### 2.3.1. Phytoene- and Phytofluene-Rich Products Tested

The tested substances consisted of a phytoene- and phytofluene-rich tomato extract in hydrogenated polydecene or squalane oil carriers (Israeli Biotechnology Research Ltd. (IBR), Yavne, Israel), each containing ca. 0.75 mg/mL phytoene and phytofluene (by UV spectrophotometry), and a phytoene- and phytofluene-rich* Dunaliella salina* extract in hydrogenated polydecene oil, containing ca. 1.0 mg/mL phytoene and phytofluene (by UV spectrophotometry).

#### 2.3.2. Experimental Conditions

The test method aims at evaluating the skin compatibility of the tested products, after a single skin application under controlled maximizing experimental conditions [29]. Each study included 10-11 subjects (subjects were healthy; male and female; 18–70 years old; all skin types; Fitzpatrick phototypes I–V). Single patch applications of the test material (applied neat, without further dilution) were made on the subjects' back, under occlusive patches (Finn Chamber, Epitest Ltd.; Trumed®; or IQ Ultra), for 48 h. Distilled water was applied under a control patch.

#### 2.3.3. Examinations and Observations

Skin compatibility of the tested substances was evaluated visually 15–30 minutes after patch removal, based on skin examination of the treated and control areas and reporting of any sensations of discomfort by the test subjects. Observed erythema was graded on a 5-point scale from “no visible erythema” to “caustic effect-erosive and/or necrotic aspect.” Skin irritation was graded according to the International Contact Dermatitis Research Group scale (from “no reaction” to “extreme positive reaction”). The appearance of other signs of cutaneous reaction was also recorded, including infiltration/edema, papules, vesicles, bulla, petechiae, fissures, desquamation, dryness, hyper- or hypopigmentation, follicular reactions, exudation and/or surface encrustation, scabs, itching, heating, burning, and any extension of reactions beyond the area of the patch. Finally, observations were collected from study participants, and any other adverse events were recorded. An individual daily irritation score (IDIS) was calculated as follows: IDIS = sum of the grades obtained for visible clinical signs observed on the application area. Additionally a mean daily irritation score (MDIS) is calculated as follows: MDIS = ∑(IDIS)/number of valid cases.

### 2.4. Topical Toxicology* In Vivo* (Human): Repeat Insult Patch Testing (HRIPT)

#### 2.4.1. Phytoene- and Phytofluene-Rich Products Tested

The tested substances consisted of a phytoene- and phytofluene-rich* Dunaliella salina* extract in hydrogenated polydecene oil, containing ca. 0.02 mg/mL phytoene and phytofluene (by UV spectrophotometry), and of a phytoene- and phytofluene-rich tomato fruit extract in squalane, containing ca. 0.75 mg/mL phytoene and phytofluene (by UV spectrophotometry) (Israeli Biotechnology Research Ltd. (IBR), Yavne, Israel).

#### 2.4.2. Experimental Conditions

The studies were performed on 50 subjects each (subjects were healthy; male and female; 18–70 years old; all skin types; Fitzpatrick phototypes I–V). The test method used was the widely recognized protocol described by Marzulli and Maibach [30]. During the first phase (induction phase), nine patch applications of 20 *μ*L of the test material (applied neat, without further dilution) were made under occlusive Finn Chamber patches (Epitest Ltd.), on the same skin site on the subjects' back, over a 3-week period. Each application lasted for 48 to 72 h. Skin reactions were scored 15–30 minutes after removal of each patch application, following which a new patch application is performed. The induction phase was followed by a 2–4 week rest phase, after which a single 48 h application (challenge phase) was carried out under the same conditions as above, both on the same skin site as used in the induction phase and on a corresponding fresh skin site on the opposite side of the subjects' back. Scoring of skin reactions was performed 30 minutes, and once more 48 hours, after removal of the challenge patch.

#### 2.4.3. Examinations and Observations

The scoring of skin reactions was performed as follows: intensity of erythema and edema was assessed on an ordinal scale from “none” to “severe”; the appearance of main signs of cutaneous reaction was noted, such as erythema, edema, papules, vesicles, bulla, scabs, petechiae, fissures, desquamation, dryness, hyper- or hypopigmentation or other discoloration, and follicular reactions. A grade was either assigned according to the International Contact Dermatitis Research Group (ICDRG) scale or calculated based on observed signs (mean daily irritation score = ∑[individual  daily  irritation  scores]/number of subjects, where individual daily irritation scores were calculated based on grading of each observed sign of reaction).

## 3. Results

### 3.1. *In Vitro* Toxicology: Cytotoxicity by Neutral Red Release (NRR) Protocol

In the NRR protocol, both the tomato extract and the* Dunaliella salina* extract were classified as having* negligible cytotoxicity*, upon exhibiting an LD50 of over 50%, and less than 20% cell death at the 20% dilution.

### 3.2. *In Vitro* Toxicology: Genotoxic Potential

The results of the test are summarized in Table 2.

According to the results shown in Table 2, the tested sample may be classified as having no genotoxic potential up to a 1/10 dilution.

### 3.3. Topical Toxicology* In Vivo* (Human): 48 h Patch Testing

The results of the studies described in Section 2.3 are summarized in Table 3.

Based on the observations made during the 48 h Patch Testing, the phytoene- and phytofluene-rich extracts tested may be classified as nonirritant and having very good skin compatibility.

### 3.4. Topical Toxicology* In Vivo* (Human): Repeat Insult Patch Testing (HRIPT)

During the induction phase, no clinically significant objective signs of skin reaction were noted in any subjects with either product tested.

With the* Dunaliella* extract, the average erythema grade was 1 (slight erythema only). During the challenge phase, once again no clinically significant objective signs of skin reaction were noted in any subjects (ICDRG grade = 0, no reaction). Overall irritation grades were recorded as 0.00 for both the induction and the challenge phase. No significant clinical adverse event was noted during the study.

With the tomato extract, the mean daily irritation score was 0 at every testing point in the study.

Therefore, it may be concluded that the tested extracts did not induce any sensitization or allergic reactions and are very well tolerated on human skin.

## 4. Discussion

The different pieces of experimental evidence presented in this paper point to the fact that the use of colorless carotenoids phytoene and phytofluene in topical formulations does not pose important safety concerns. Apart from this, there are other pieces of evidence indicating that these compounds are part of the common diets of many ethnicities, that they are present in human fluids and tissues, and that they may even provide health and cosmetic benefits, as summarized in the introduction. In relation to the presumable safety of phytoene and phytofluene it is to be noted that there is ample evidence concerning the safety of the other major carotenoids found in humans and in amounts much higher than those commonly achievable through the diet.

### 4.1. Dietary Sources of Phytoene and Phytofluene

Almost without exception, phytoene and phytofluene levels in foods have not been included in food composition tables or databases [31–36] or in dedicated studies [9, 37, 38]. However, this situation is expected to change in the near future as evidence is accumulating that phytoene and phytofluene can provide health benefits [26, 39], which is expected to lead to heightened interest in these compounds.

However, phytoene and phytofluene can be found in common foods in Western diets like apricot, cantaloupe, banana, carrot, melon, oranges, peppers, watermelon, lemon, clementines, grapefruits, avocado, mandarin, nectarine, peach, and a wide variety of tomatoes and derivatives (reviewed in [26]) and in other more exotic and highly nondomesticated foods. Examples of the latter would be caja [40], buriti, mamey, marimari, physalis [41], or gac [42], among others. It is noteworthy that phytoene and phytofluene are also found in ancient Andean wild relatives of the domesticated tomato [43]. Thus, it can be inferred that phytoene and phytofluene are common in the diet of humans from different geographical regions and ethnicities and that it is sensible to think that they have been part of their diet for centuries if not millennia.

### 4.2. Phytoene and Phytofluene Intakes

The carotenoid intakes reported in different countries are summarized in Table 4.

Phytoene and phytofluene have not been taken into account in most dietary intake studies. Recently, these were estimated to be 2.0 mg and 0.7 mg, respectively, in Luxembourg [6]. The fact that the intakes of phytoene were second only to *α*- + *β*-carotene and that these intakes were higher than those of lycopene (1.8 mg) is noteworthy. Higher intakes of phytoene relative to lycopene may also be expected in other countries. Phytoene and phytofluene typically accompany lycopene in the dietary sources of the latter (e.g., tomato and derivatives, watermelons, and red-fleshed citrus), although their levels are normally considerably lower [9, 44–47]. However, the distribution of the colorless carotenoids among dietary sources seems to be broader as these compounds are also found in widely consumed fruits and vegetables that do not contain detectable levels of lycopene, like most carrots, peaches, nectarines, red peppers, oranges, and other citrus varieties [26].

### 4.3. Safety of Dietary Carotenoids

Being naturally biosynthesized by all plants, it can be claimed that carotenoids have always been present in the diet of the human species. Thus, all green leaves and other plant photosynthetic tissues contain high amounts of lutein and the provitamin A *β*-carotene [48], two carotenoids bioavailable in humans that are thought to provide health benefits [2, 49]. Likewise, other plant foods (roots, fruits, and seeds) provide other health-promoting carotenoids like lycopene, *β*-cryptoxanthin, zeaxanthin, phytoene, and phytofluene [26, 39, 50–55]. Interestingly, the first natural food of newborns, their mothers' milk, contains carotenoids, including phytoene and phytofluene, with high levels of lutein, which is thought to be important for their development [49, 56]. Phytoene and phytofluene have also been reported in plasma at concentrations within the range 0-1 *μ*M [11, 19, 57–60] and in several human tissues (liver, lung, breast, cervix, prostate, colon, and skin) at levels in the range of ng/g [13]. Taking all these facts into consideration it can be presumed that carotenoids at dietary levels are safe and that these compounds could even provide diverse health benefits.

#### 4.3.1. Safety Data of Other Major Dietary Carotenoids

There is ample evidence that *β*-carotene from diverse sources (synthetic, plants, algae, and fungi) is a safe compound. LD_50_ values up to over 20000 mg/kg and No-Observed-Adverse-Effect Level (NOAEL) from 696 up to 4175 mg/kg body weight/day have been reported. The EFSA Panel on Food Additives and Nutrient Sources added to food stated that “the use of *β*-carotene [E 160a (ii)] as food color [is] not of safety concern, provided that the estimated combined intake from their use as a food additive and as food supplement, is not more than the amount likely to be ingested as a result of the regular consumption of the foods in which they occur naturally (5–10 mg/day).” [61].

Values of LD_50_ over 2000 mg/kg and NOAEL between 200 and 1000 mg/kg body weight/day have been reported for lutein. Moreover, the EFSA Panel on Food Additives and Nutrient Sources added to Food established an ADI of 1 mg/kg body weight/day [62]. Several lutein products have been categorized as GRAS by the FDA [63–68].

Values of LD_50_ over 3000 mg/kg body weight and of NOAEL ranging from 50 to 600 mg/kg body weight/day have been reported for lycopene. The Panel on Food Additives, Flavorings, Processing Aids, and Materials in Contact with Food derived an ADI of 0.5 mg/kg body weight/day for lycopene from all sources. The panel also stated that the ADI applies to lycopene from tomatoes, [69]. The EFSA Panel on Dietetic Products, Nutrition and Allergies also provided an opinion on the safety of a lycopene oleoresin from non-GM high-lycopene tomatoes. This product contained 5–15% lycopene, phytoene (0.5–0.75%), phytofluene (0.4–0.65%), and *β*-carotene (0.2–0.35%), as well as other lipophilic compounds, including tocopherols (1.5–3%). The panel stated that the lycopene oleoresin was as safe as lycopene from other accepted sources [70]. Several lycopene or lycopene-rich products have been categorized as GRAS by the FDA, including synthetic lycopene [71], tomato lycopene [71], tomato pulp powder [72], concentrated tomato lycopene extract [73], and even lycopene produced by* Escherichia coli *expressing lycopene biosynthetic enzymes [74].

Values of LD_50_ over 8000 mg/kg [75] and NOAEL of 150 mg/kg body weight per day have been reported for synthetic zeaxanthin. The EFSA Panel on Dietetic Products, Nutrition and Allergies has concluded that daily intakes of 0.75 mg/kg body weight do not raise safety concerns [76]. Several zeaxanthin products have been categorized as GRAS by the FDA, including zeaxanthin from* Capsicum annuum* [77] and marigold* (Tagetes erecta)* [78].

## 5. Conclusions

Carotenoids are natural compounds widespread in foods that are naturally present in the diet and can be used as additives or ingredients of supplements. They are present in human plasma and tissues. There is ample toxicological evidence concerning their safety, and indeed there are carotenoids or carotenoid-containing products categorized as GRAS by the US FDA. On the other hand, EFSA has established ADIs of 0.5 and 1 mg/kg body weight/day for the common dietary carotenoids lycopene and lutein, respectively. This means that daily intakes of 30 and 60 mg of such carotenoids by a person of 60 kg are not considered of safety concern. These intakes are considerable higher than the daily total dietary carotenoid intakes assessed in several countries (ranging from ca. 7.5 to 21 mg/day, Table 2).

The colorless carotenoids phytoene and phytofluene are present in widely consumed foods (like tomatoes, citrus, or carrots among many others) as well as in human plasma, milk, internal tissues, and skin. Some studies suggest that phytoene and phytofluene may be involved in diverse health-promoting actions. Thus, phytoene and phytofluene could act as antioxidants or anti-inflammatory compounds or protect against cancer and light-induced damage in the skin. Being naturally present in common and exotic foods, including ancient wild relatives of the domesticated tomato, their intake at dietary levels is not expected to be of safety concern. However, since their interest in the context of the diet, functional foods, and nutricosmetics is expanding, it is important to conduct studies aimed at assessing their safety.

Thus, we present herein the results of* in vitro* cytotoxicity studies (NRR protocol) on phytoene- and phytofluene-rich extracts from two sources (tomato and* Dunaliella salina*), showing that these extracts have negligible cytotoxicity in this model; by an* in vitro* genotoxicity study on a phytoene- and phytofluene-rich extract of* Dunaliella salina*, showing no genotoxic potential for this extract in the tested model; by* in vivo* (human) 48 h patch tests of three phytoene- and phytofluene-rich extracts from two sources (tomato and* Dunaliella salina*), showing that these extracts have very good skin compatibility; and by a longer-term Human Repeat Insult Patch Test of a phytoene- and phytofluene-rich* Dunaliella salina* extract, showing that the tested extract did not induce any skin sensitization and was very well tolerated on human skin.

The facts that both EFSA and FDA have provided positive opinions concerning the safety of tomato products and that these contain phytoene and phytofluene at lower amounts relative to lycopene but still high, considering the classification of Britton and Khachik [26, 79], adds support to the safety of phytoene and phytofluene as food ingredients. The above data speak strongly in favor of the safe use of phytoene- and phytofluene-rich products for a variety of human uses. In this light and that of other data on the safety of other dietary carotenoids, it would not be surprising that ADIs comparable to those established for lycopene or lutein could eventually be established for phytoene and phytofluene, although, of course, the pertinent toxicological studies will need to be performed.

## Figures and Tables

**Table 1:** Classification of cytotoxicity results in the NRR method.

IC50 (%)	% cell death at the 50% dilution	Classification
>50	<20	Negligible cytotoxicity
	>20	Low cytotoxicity
>25 and <50		Moderate cytotoxicity
<25		High cytotoxicity

**Table 2:** Genotoxicity results.

Test substance	Dilution/concentration	Repair ratio
Phytoene- and phytofluene-rich *Dunaliella* extract	1/10	0.92
	1/100	1.11
	1/1000	1.18
	1/10000	1.05

MMS	10 mM	6.26
	2 mM	2.44

**Table 3:** 48 h patch test results.

Test substance	Tomato extract in squalane	Tomato extract in hydrogenated polydecene	*Dunaliella* extract in hydrogenated polydecene
Reactions noted	None	None	None
# reactive subjects (%)	0 (0%)	0 (0%)	0 (0%)
MDIS	0	0	0
Conclusion	Very good skin compatibility/nonirritant	Very good skin compatibility/nonirritant	Very good skin compatibility/nonirritant

**Table 4:** Reported carotenoid intakes (mg/day) in different countries. Adapted from Maiani et al. (2009) and Biehler et al. (2012).

Country	TI^*∗*^	L + Z	BCR	LYC	ACA	BCA	PT	PTF
Germany	9.37	4.87	0.28	0.75	0.28	3.09	n.r.	n.r.
Denmark	10.10	5.25	0.40	0.71	0.30	3.43	n.r.	n.r.
Italy	15.73	7.09	0.63	2.36	0.47	5.20	n.r.	n.r.
Sweden	7.52	3.61	0.38	0.83	0.23	2.41	n.r.	n.r.
UK	8.65	4.33	0.35	0.78	0.26	2.86	n.r.	n.r.
Greece	20.97	8.39	0.63	4.40	0.84	6.71	n.r.	n.r.
France	13.98	6.99	0.56	1.26	0.42	4.75	n.r.	n.r.
Netherlands	8.76	4.21	0.44	0.88	0.26	2.89	n.r.	n.r.
Spain	12.79	5.76	0.51	1.79	0.38	4.35	n.r.	n.r.
Luxembourg	15.30	1.80	1.40	1.80		7.60^*∗∗*^	2.00	0.70

L: lutein; Z: zeaxanthin; BCR: *β*-cryptoxanthin; LYC: lycopene; ACA: *α*-carotene; BCA: *β*-carotene; PT: phytoene; PTF: phytofluene; n.r.: not reported; ^*∗*^total Intake (TI): sum of the individual values of L + Z, BCR, LYC, ACA, and BCA (and also of the levels of PT and PTF in the case of Luxembourg); ^*∗∗*^sum of the intakes of ACA + BCA.
